# Supplementary material for: Persistent TFIIH binding to non-excised DNA damage causes cell and developmental failure
Source: Nat Commun. 2024 Apr 25;15:3490. doi: 10.1038/s41467-024-47935-9 (PMC11045817; doi:10.1038/s41467-024-47935-9)
Supplement: Supplementary file 1 — Supplementary Information [file 41467_2024_47935_MOESM1_ESM.pdf]

Supplementary Materials for

Persistent TFIIH binding to non-excised DNA damage causes cell and developmental failure

Alba Muniesa-Vargas[1,3], Carlota Davó-Martínez[1,3], Cristina Ribeiro-Silva[1,3], Melanie van der Woude[1], Karen L. Thijssen[1], Ben Haspels[2], David Häckes[1], Ülkem U. Kaynak[1], Roland Kanaar[2], Jurgen A. Marteijn[2], Arjan F. Theil[1], Maayke M. P. Kuijten[2], Wim Vermeulen[1], Hannes Lans[1,*]

1. Department of Molecular Genetics, Erasmus MC Cancer Institute, Erasmus University Medical Center, 3015 GD, Rotterdam, The Netherlands

2. Department of Molecular Genetics, Erasmus MC Cancer Institute, Oncode Institute, Erasmus University Medical Center, 3015 GD, Rotterdam, The Netherlands

3. These authors contributed equally

*Correspondence: w.lans@erasmusmc.nl

This PDF file includes:

Supplementary Text

Supplementary Figures 1 to 3

Supplementary Tables 1 to 4

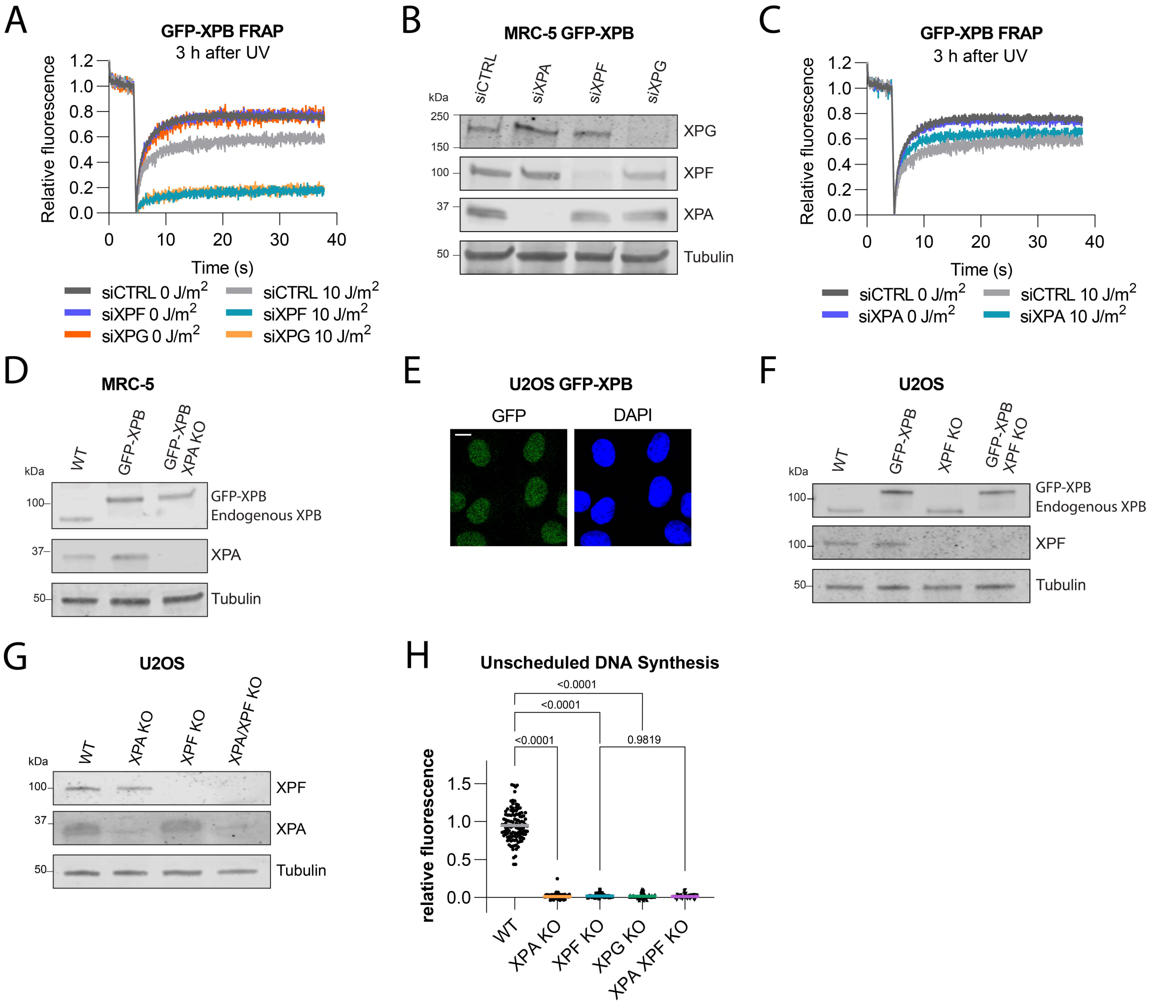

Supplementary Figure 1. FRAP, UDS and immunoblot analyses of knock-in and knock-out cell lines. (A) FRAP of GFP-tagged XPB in MRC-5 cells treated with control (CTRL), XPF or XPG siRNA, non-irradiated (0 J/m^2) or 3 h after UV (10 J/m^2); Each FRAP curve represents the average of three independent experiments. Quantification and number of cells are shown in Figure 1C and legend. **(B)** Immunoblot of GFP-XPB MRC-5 cells treated with control, XPA, XPF or XPG siRNA, stained with antibodies against XPG, XPF, XPA and tubulin (loading control). **(C)** FRAP of GFP-tagged XPB in MRC-5 cells treated with control or XPA siRNA, non-irradiated (0 J/m^2) or 3 h after UV (10 J/m^2); Each FRAP curve represents the average of three independent experiments. Quantification and number of cells are shown in Figure 1C and legend. **(D)** Immunoblot of wild type (WT) and GFP-XPB knock-in MRC-5 cells, proficient for XPA or with XPA knockout (KO), stained with antibodies against XPB, tubulin (loading control) and XPA. **(E)** Representative immunofluorescence image of U2OS GFP-XPB knock-in cells. DNA was stained with DAPI. Scale bar, 10 µM. **(F)** Immunoblot of wild type (WT) and XPF knockout (KO) U2OS cells without or with GFP-XPB knock-in, stained with antibodies against XPB, XPF and tubulin (loading control). **(G)** Immunoblot of wild type (WT), XPA knockout (KO), XPF KO and double XPA/XPF KO U2OS cells, stained with antibodies against XPF, XPA and tubulin (loading control). **(H)** Unscheduled DNA synthesis analysis of wild type (WT), XPA knockout (KO; also expressing GFP-XPB), XPF KO, XPG KO and double XPA/XPF KO U2OS cells, irradiated with 16 J/m^2 UV. Mean with SEM of three independent experiments. Numbers represent p-values (ANOVA corrected for multiple comparisons). *n* (left to right)=119, 172, 172, 117, 105. Immunoblots and immunofluorescence experiments were replicated with similar results. Source data are provided as a Source Data file.

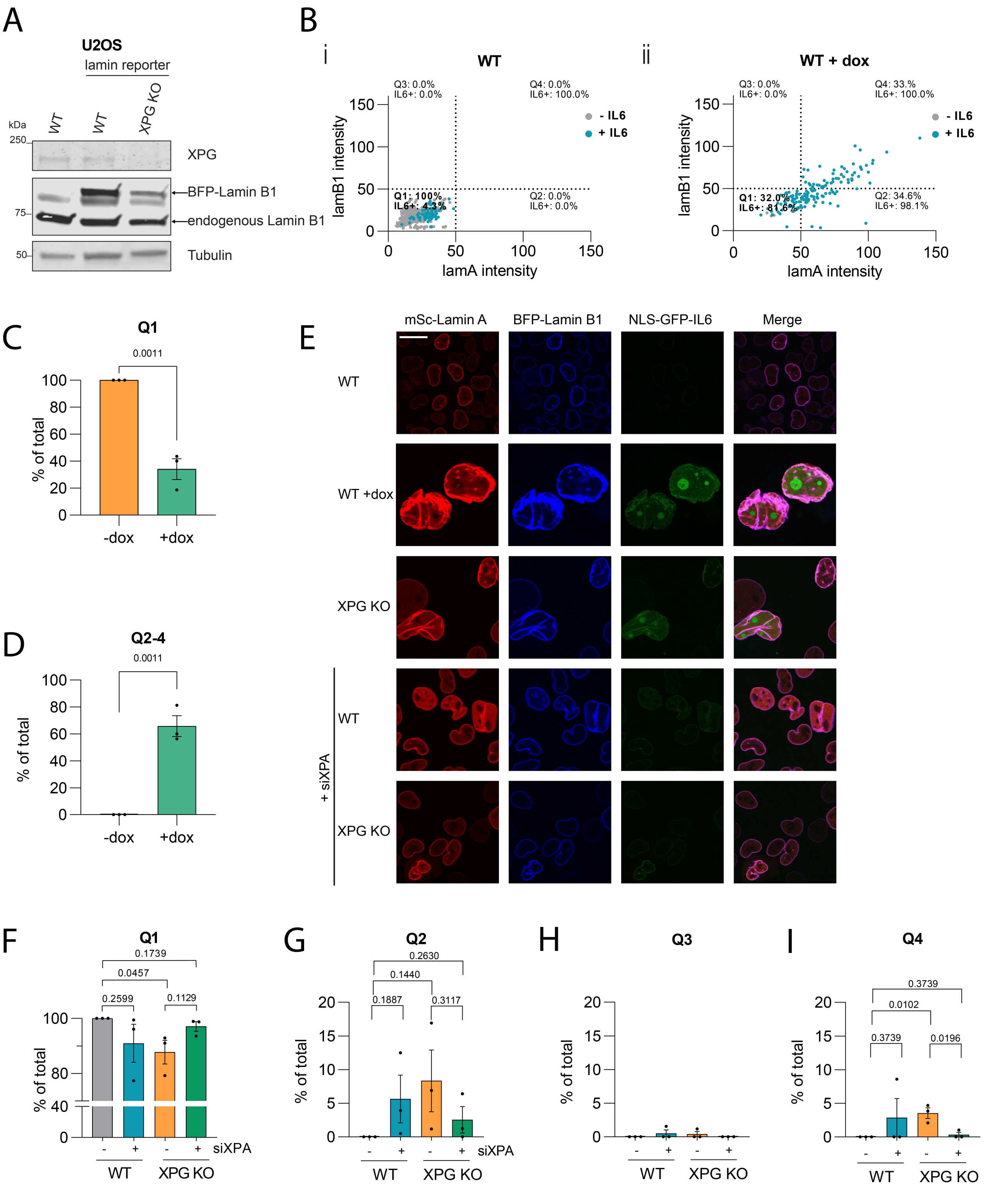

Supplementary Figure 2. Lamin and IL-6-based senescence reporter plasmid. (A) Immunoblot of control and transgenic wild type (WT) and XPG knockout (KO) cells stably expressing the lamin-based senescence reporter plasmid, stained with antibodies against XPG, lamin B1 and tubulin (loading control). Immunoblot was replicated with similar result. **(B)** Scatter plot showing relative mScarlet-Lamin A (lamA) and BFP-Lamin B1 (lamB1) intensities in wild type (WT) U2OS cells (i) without treatment or (ii) after 2 h 1 µM doxorubicin treatment (+dox). Each dot represents a cell. Blue color indicates senescence-associated induction of GFP-IL6. Grey color indicates no GFP-IL6 expression. Scatter plot is divided in four quadrants (Q1-Q4) and percentage of cells in each quadrant and percentage of IL6 positive cells (IL6+) is indicated per quadrant. *n*=2109 for WT and *n*=153 for WT +dox. Cells from three replicate experiments are shown. **(C and D)** Graphs showing average percentage (with SEM) of cells in Q1 or in Q2, Q3 and Q4 (Q2-4) from three independent replicate experiments, after no treatment (-dox) or 2 h treatment of 1 µM doxorubicin. **(E)** Representative images of untreated wild type (WT), doxorubicin-treated WT (+dox), untreated XPG knockout (KO) and siXPA-treated WT and XPG KO U2OS cells stably expressing the mScarlet-Lamin A/BFP-Lamin B1/GFP-IL6 senescence reporter plasmid. Scale bar, 25 µm. **(F-I)** Graphs showing the average percentage (with SEM) of WT or XPG KO U2OS cells, without or with siXPA treatment, in Q1 (F), Q2 (G), Q3 (H) or Q4 (I), from three independent replicate experiments. Numbers represent p-values (unpaired two-sided t-test). Source data are provided as a Source Data file.

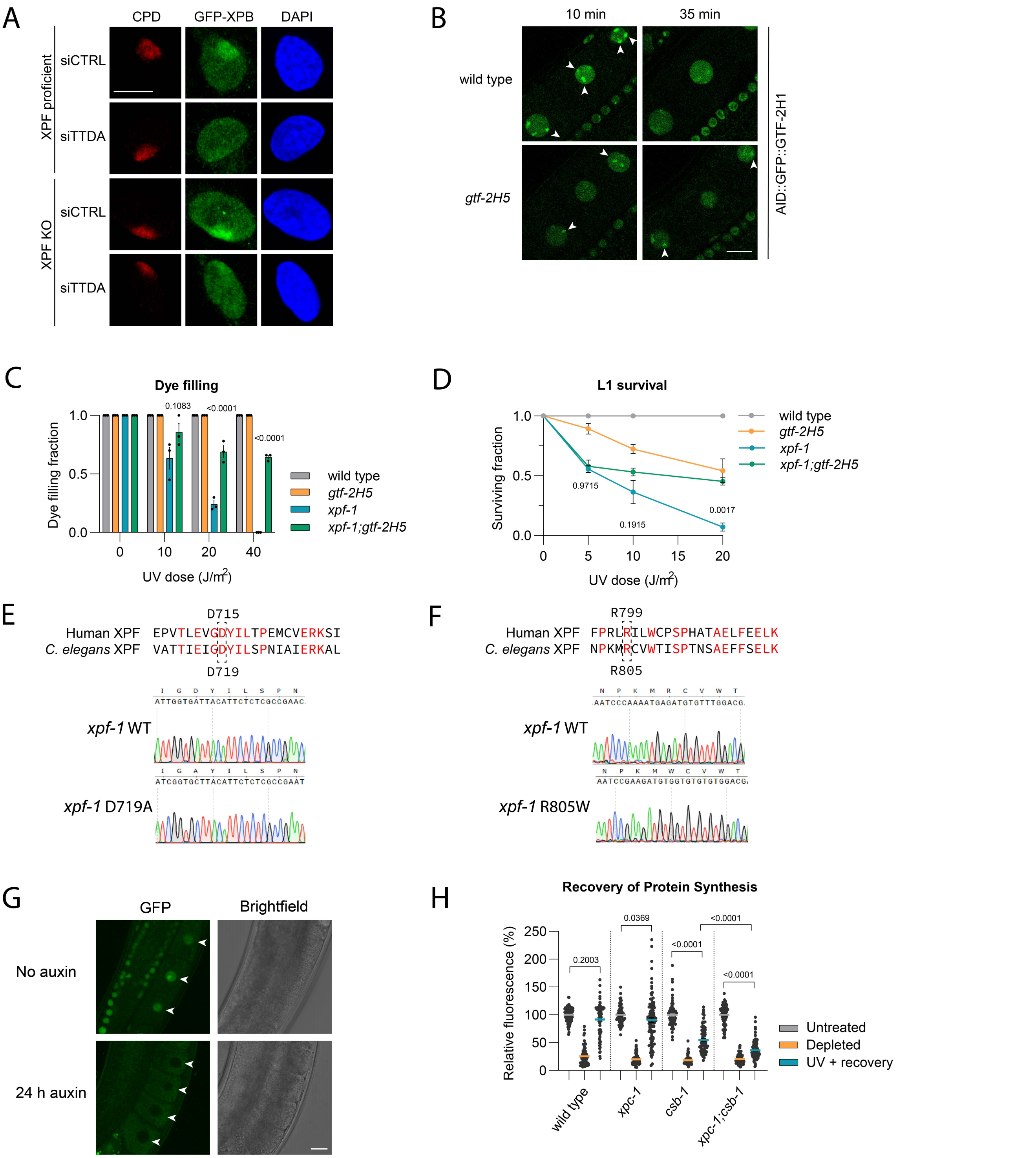

Supplementary Figure 3. TTDA/GTF-2H5-dependent prolonged TFIIH binding to DNA damage impairs neuron functionality in *C. elegans*. (A) Immunofluorescence of XPF proficient and knockout (KO) U2OS GFP-XPB cells, treated with control or TTDA siRNA, 30 min after 60 J/m^2 UV-C through a microporous filter to induce LUD. Images show GFP-XPB accumulation at LUD marked by anti-CPD staining. DNA is stained with DAPI. Scale bar, 10 µm. Immunofluorescence was replicated with similar result **(B)** Recruitment of AID-GFP-tagged GTF-2H1 to UV-B-damaged chromosomes in *C. elegans* oocytes (arrowheads) of living wild type or *gtf-2H5* animals, 15 and 35 minutes after 300 J/m^2 UV-B irradiation. Scale bar, 10 µm. **(C)** Fraction of dye filling wild type, *xpf-1*, *gtf-2H5* and *xpf-1;gtf-2H5* animals, 72 h after different UV-B dose. Mean with SEM of three independent experiments. Numbers represent p-values (ANOVA corrected for multiple comparisons). *n* (left to right)=62, 55, 63, 62, 70, 32, 56, 51, 61, 36, 50, 46, 57, 31, 37, 60. **(D)** L1 larvae survival assays after UV-B irradiation with wild type, *xpf-1*, *gtf-2H5* and *xpf-1;gtf-2H5* animals. Mean with SEM of three independent experiments. Numbers represent p-values (ANOVA corrected for multiple comparisons). *n* (left to right)= WT:1000, 923, 857, 831; *xpf-1:*1100, 911, 879, 791; *gtf-2H5:*1050, 1594, 1377, 1412; *xpf-1;gtf-2H5:*1000, 1224, 954, 913. **(E)** Top panel shows alignment of a short fragment of human XPF with *C. elegans* XPF-1, indicating the *C. elegans* residue 719 that is changed from aspartic acid to alanine in the *xpf-1* D719A mutant. Lower panel shows the DNA sequence of the corresponding genomic region in wild type (WT) and *xpf-1* D719A mutants. **(F)** Top panel shows alignment of a short fragment of human XPF with *C. elegans* XPF-1, indicating the *C. elegans* residue 805

that is changed from arginine to tryptophan in the *xpf-1* R805W mutant. Lower panel shows the DNA sequence of the corresponding genomic region in wild type (WT) and *xpf-1* R805W mutants. **(G)** AID-GFP-tagged GTF-2H1 levels in oocyte and germ cell nuclei (arrowheads) of animals cultured without auxin or 24 h in the presence of 100 µM auxin. Scale bar, 10 µm. **(H)** Recovery of Protein Synthesis (RPS) assay in wild type, *xpc-1*, *csb-1* and *xpc-1;csb-1* animals. Shown are the AID::GFP fluorescence levels in head muscle cells of animals either untreated, after culturing for 2 h on 100 µM auxin ('Depleted') or after culturing on 100 mM auxin for 2 h and then irradiated with 120 J/m^2 UV-B and left to recover for 48 h ('UV + recovery'). Average values of from three independent experiments. Numbers represent p-values (ANOVA corrected for multiple comparisons). *n* (left to right)= 95, 91, 86, 104, 100, 101, 88, 88, 102, 93, 96, 110. Source data are provided as a Source Data file.

Supplementary Table 1. Cell lines

Cell line	Genotype	Reference
MRC-5	GFP-XPB	Ribeiro-Silva *et al.*, 2020
MRC-5	GFP-XPB / XPA KO	*This study*
U2OS wild type	-	-
U2OS	GFP-XPB	*This study*
U2OS	GFP-XPB / XPF KO	*This study*
U2OS	XPA KO	*This study*
U2OS	XPF KO	Sabatella *et al.*, 2018
U2OS	XPA KO XPF KO	*This study*
U2OS	GFP-XPB XPF-mCherry	*This study*
U2OS	GFP-XPB XPF(P379S)-mCherry	*This study*
U2OS	GFP-XPB XPF(R799W)-mCherry	*This study*
U2OS	GFP-XPB XPF(C236R)-mCherry	*This study*
U2OS	GFP-XPB XPA KO	*This study*
U2OS	BFP-Lamin B1 mScarlet-Lamin A	*This study*
U2OS	XPG KO / BFP-Lamin B1 mScarlet-Lamin A	*This study*
U2OS	CSB-mClover	Llerena Schiffmacher *et al.,* 2023

Supplementary Table 2. Primary antibodies

Antibody	Host	Source	Dilution	
			WB	IF
CPD	Ms	MBL international, TDM-2	N.A.	1/1000
H1.2	Rb	Abcam, ab17677	1/1000	N.A.
Lamin B1	Rb	Abcam, ab16048	1/1000	N.A
Tubulin	Ms	Sigma Aldrich, B512	1/10000	N.A.
XPA	Rb	GeneTex, GTX103168	1/2000	N.A.
XPB	Rb	Abcam, Ab190698	1/1000	1/1000
XPC	Rb	Bethyl, A301-121A	1/2000	N.A.
XPD	Ms	Abcam, ab54676	1/1000	N.A.
XPF	Ms	Santa Cruz, sc-136153	1/500	N.A.
XPG	Rb	Bethyl, A301-484A	1/1000	N.A.

Supplementary Table 3. Secondary antibodies

Antibody	Host	Source	Dilution	
			WB	IRDye
Rabbit	Goat	Sigma, sab4600215	1/10000	770
Rabbit	Goat	Sigma, sab4600200	1/10000	680
Mouse	Goat	Sigma, sab4600199	1/10000	680
Mouse	Goat	Sigma, sab4600214	1/10000	770
**Antibody**	**Host**	**Source**	**IF**	**AlexaFluor**
Rabbit	Goat	Invitrogen, A-11034	1/1000	488
Mouse	Goat	Invitrogen, A-21424	1/1000	555

Supplementary Table 4. *C. elegans* strains

Strain	Genotype
GJ1553	*xpc-1(tm3886) IV*
GJ1564	*xpf-1(tm2842) II*
GJ1566	*xpa-1(ok698) I*
GJ2501	*xpg-1(tm1682) I*
HAL26	*csb-1(ok2335) X*
HAL94	*gtf-2H5(tm6360) III*
HAL100	*xpf-1(emc57[xpf-1::GFP]) II*
HAL241	*xpf-1(tm2842) II; emcSi70[P(unc-119)::TIR::mRuby] gtf-2H1(emc202[AID::GFP:: gtf-2H1]) IV*
HAL404	*xpf-1(emc105*[R805W]) *emc57[xpf-1::GFP] II*
HAL407	*xpa-1(ok698) I; xpf-1(tm2842) II*
HAL409	*xpg-1(tm1670) xpa-1(ok698) I*
HAL410	*xpf-1(tm2842) II; gtf-2H5(tm6360) III*
HAL412	*xpf-1(tm2842) II; xpc-1(tm3886) IV*
HAL413	*xpg-1(tm1670) I; xpc-1(tm3886) IV*
HAL414	*xpf-1(tm2842) II; csb-1(ok2335) X*
HAL415	*xpg-1(tm1670) I; csb-1(ok2335) X*
HAL416	*xpa-1(ok698) I; emcSi70[P(unc-119)::TIR::mRuby] gtf-2H1(emc202[AID::GFP:: gtf-2H1]) IV*
HAL417	*xpa-1(ok698) I; xpf-1(tm2842) II; emcSi70[P(unc-119)::TIR::mRuby] gtf-2H1(emc202[AID::GFP:: gtf-2H1]) IV*
HAL418	*xpf-1(tm2842) II; xpc-1(tm3886) IV; csb-1(ok2335) X*
HAL504	*gtf-2H1(emc202[AID::GFP:: gtf-2H1]) IV*
HAL526	*ieSi57 [P(eft-3)::TIR1::mRuby] II; ieSi58 [P(eft-3)::AID::GFP] IV; csb-1(ok2335) X*
HAL534	*ieSi57 [P(eft-3)::TIR1::mRuby] II; ieSi58 [P(eft-3)::AID::GFP] xpc-1(tm3886) IV*
HAL535	*ieSi57 [P(eft-3)::TIR1::mRuby] II; ieSi58 [P(eft-3)::AID::GFP] IV; xpc-1(tm3886) IV; csb-1(ok2335) X*
HAL805	*xpf-1(emc98*[D719A]) *emc57[xpf-1::GFP]II*
CA1202	*ieSi57 [P(eft-3)::TIR1::mRuby] II; ieSi58 [P(eft-3)::AID::GFP] IV*
