# Supplementary material for: Persistent TFIIH binding to non-excised DNA damage causes cell and developmental failure
Source: Nat Commun. 2024 Apr 25;15:3490. doi: 10.1038/s41467-024-47935-9 (PMC11045817; doi:10.1038/s41467-024-47935-9)
Supplement: Supplementary file 3 — Reporting Summary [file 41467_2024_47935_MOESM3_ESM.pdf]

nature portfolio

Corresponding author(s): Hannes Lans

Last updated by author(s): Mar 25, 2024

Reporting Summary

Nature Portfolio wishes to improve the reproducibility of the work that we publish. This form provides structure for consistency and transparency in reporting. For further information on Nature Portfolio policies, see our Editorial Policies and the Editorial Policy Checklist.

Statistics

For all statistical analyses, confirm that the following items are present in the figure legend, table legend, main text, or Methods section.

n/a	Confirmed	
☐	☒	The exact sample size (*n*) for each experimental group/condition, given as a discrete number and unit of measurement
☐	☒	A statement on whether measurements were taken from distinct samples or whether the same sample was measured repeatedly
☐	☒	The statistical test(s) used AND whether they are one- or two-sided<br>*Only common tests should be described solely by name; describe more complex techniques in the Methods section.*
☒	☐	A description of all covariates tested
☒	☐	A description of any assumptions or corrections, such as tests of normality and adjustment for multiple comparisons
☐	☒	A full description of the statistical parameters including central tendency (e.g. means) or other basic estimates (e.g. regression coefficient) AND variation (e.g. standard deviation) or associated estimates of uncertainty (e.g. confidence intervals)
☐	☒	For null hypothesis testing, the test statistic (e.g. *F*, *t*, *r*) with confidence intervals, effect sizes, degrees of freedom and *P* value noted<br>*Give P values as exact values whenever suitable.*
☒	☐	For Bayesian analysis, information on the choice of priors and Markov chain Monte Carlo settings
☒	☐	For hierarchical and complex designs, identification of the appropriate level for tests and full reporting of outcomes
☒	☐	Estimates of effect sizes (e.g. Cohen's *d*, Pearson's *r*), indicating how they were calculated

Our web collection on statistics for biologists contains articles on many of the points above.

Software and code

Policy information about availability of computer code

Data collection	Microscopy data was obtained using commercially available Leica LAS AF or Carl Zeiss LSM software.
Data analysis	Data was analyzed by Leica LAS AF (version 2.7.4.10100) and LAS X software (version 3.5.6.21594), Carl Zeiss LSM (version 14.0.0.0), ImageJ/ Fiji software (version 1.52p) and further processed in Excel (2016). Image Studio Lite (Version 5.2.5) was used for Western blot acquisition and analysis Data was plotted and analyzed using GraphPad Prism (version 9.4.0).

For manuscripts utilizing custom algorithms or software that are central to the research but not yet described in published literature, software must be made available to editors and reviewers. We strongly encourage code deposition in a community repository (e.g. GitHub). See the Nature Portfolio guidelines for submitting code & software for further information.

Data

Policy information about availability of data

All manuscripts must include a data availability statement. This statement should provide the following information, where applicable:

- Accession codes, unique identifiers, or web links for publicly available datasets
- A description of any restrictions on data availability
- For clinical datasets or third party data, please ensure that the statement adheres to our policy

A data availability statement is included in the manuscript: Source data underlying figures 1–5 and all supplementary figures are provided as Source Data files with this paper. Any other data are available from the corresponding author upon reasonable request..

Research involving human participants, their data, or biological material

Policy information about studies with human participants or human data. See also policy information about sex, gender (identity/presentation), and sexual orientation and race, ethnicity and racism.

Reporting on sex and gender	not applicable
Reporting on race, ethnicity, or other socially relevant groupings	not applicable
Population characteristics	not applicable
Recruitment	not applicable
Ethics oversight	not applicable

Note that full information on the approval of the study protocol must also be provided in the manuscript.

Field-specific reporting

Please select the one below that is the best fit for your research. If you are not sure, read the appropriate sections before making your selection.

☒ Life sciences ☐ Behavioural & social sciences ☐ Ecological, evolutionary & environmental sciences

For a reference copy of the document with all sections, see nature.com/documents/nr-reporting-summary-flat.pdf

Life sciences study design

All studies must disclose on these points even when the disclosure is negative.

Sample size	No sample size calculation was performed, sample sizes are similar as to what is common in the field, e.g. PMID: 34108662, 34824371, 32985517 , 26151477, 20463888 and are based on the different experimental procedures e.g. technical difficulty, variation of experiments
Data exclusions	No samples were excluded.
Replication	All experiments were replicated and replications were succesful. All FRAP, dye filling and survival experiments were replicated at least three times, as indicated in the legends, unless otherwise indicated in the legends. Immublot and immunofluorescence is replicated at least twice, as indicated in the legends.
Randomization	Randomization not relevant as this study does not involve test subjects.
Blinding	Data analyses were performed by software or algorithms and therefore in an unbiased manner, making blinding therefore not applicable.

Reporting for specific materials, systems and methods

We require information from authors about some types of materials, experimental systems and methods used in many studies. Here, indicate whether each material, system or method listed is relevant to your study. If you are not sure if a list item applies to your research, read the appropriate section before selecting a response.

Materials & experimental systems

n/a	Involved in the study	
☐	☒	Antibodies
☐	☒	Eukaryotic cell lines
☒	☐	Palaeontology and archaeology
☐	☒	Animals and other organisms
☒	☐	Clinical data
☒	☐	Dual use research of concern
☒	☐	Plants

Methods

n/a	Involved in the study	
☒	☐	ChIP-seq
☒	☐	Flow cytometry
☒	☐	MRI-based neuroimaging

Antibodies

Antibodies used	Antibodies used are listed in Table S2. These are against CPD (MBL international, TDM-2:1:1000); H1.2 (Abcam, ab17677, 1:1000); Lamin B1 (Abcam, ab16048, 1:1000), Tubulin (Sigma Aldrich, B512, 1:10000); XPA (GeneTex, GTX103168, 1:2000); XPB (Abcam,

	Ab190698, 1:1000); XPC (Bethyl, A301-121A, 1:2000); XPD (Abcam, ab54676, 1:1000); XPF (Santa Cruz, sc-136153, 1:500); XPG (Bethyl, A301-484A, 1:1000)
Validation	Antibodies were validated as indicated on their manufacturer's website, in previous publications of our lab by siRNA/KO experiments or were checked by western blot or immunofluorescence in this manuscript, mostly with a siRNA/KO as control for specificity. All the antibodies used in the manuscript showed bands of expected size.<br><br>CPD (MBL international, TDM-2) verified by many previous papers from our lab and other, e.g. PMID 30165384<br>H1.2 (Abcam, ab17677) verified in PMID 32184266<br>Lamin B1 (Abcam, ab16048) verified in Fig S2A<br>Tubulin (Sigma Aldrich, B512) commonly used a loading control in the lab, verified by specific and intense band at correct height<br>XPA (GeneTex, GTX103168) verified in PMID: 53750669 and in Fig S1B and D<br>XPB (Abcam, Ab190698) verified in PMID: 33854616 and Fig S1D<br>XPC (Bethyl, A301-121A) verified in PMID: 32985517<br>XPD (Abcam, ab54676) verified in PMID 30165384<br>XPF (Santa Cruz, sc-136153) verified in Fig S1B<br>XPG (Bethyl, A301-484A, 1:1000) verified in Fig S1B

Eukaryotic cell lines

Policy information about cell lines and Sex and Gender in Research

Cell line source(s)	Cell lines and sources are listed in Table S1. U2OS and MRC-5 cells were used in previous publications (e.g Ribeiro, Nature Comm, 2020) and obtained from ATCC. GFP-XPB MRC-5 cells were from PMID 32985517. XPF KO U2OS cells were from PMID 30165384. U2OS CSB-mClover cells were from PMID 37716192. MRC-5 GFP-XPB XPA KO; U2OS GFP-XPB; GFP-XPB XPF KO; XPA KO; XPA XPF KO; GFP-XPB XPF-mCherry; GFP-XPB XPF(P379S)-mCherry; GFP-XPB XPF(R799W)-mCherry; GFP-XPB XPF(C236R)-mCherry; GFP-XPB XPA KO; BFP-Lamin B1 mScarlet-Lamin A; XPG KO / BFP-Lamin B1 mScarlet-Lamin A were generated in this study.
Authentication	WT cells were not authenticated. All knock-in and knock out cells were authenticated by genotyping PCR, western blot and/or immunofluorescence.
Mycoplasma contamination	All cell lines were routinely tested for mycoplasma and were all negative.
Commonly misidentified lines<br>(See ICLAC register)	No commonly misidentified cell lines were used in the study.

Animals and other research organisms

Policy information about studies involving animals; ARRIVE guidelines recommended for reporting animal research, and Sex and Gender in Research

Laboratory animals	C. elegans strains used are listed in Table S4. These are strains wild type (N2 bristol) and GJ1553, GJ1564, GJ1566, GJ2501, HAL26, HAL94, HAL100, HAL241,HAL404,HAL407, HAL409, HAL410, HAL412, HAL413,HAL414,HAL415, HAL416,HAL417, HAL418,HAL504,HAL526,HAL534,HAL535, HAL805, CA1202. C. elegans between 0 day old to 8 days old were used.
Wild animals	This study did not involve wild animals.
Reporting on sex	C. elegans is hermafrodite
Field-collected samples	No samples were collected from the field.
Ethics oversight	Not applicable for C. elegans

Note that full information on the approval of the study protocol must also be provided in the manuscript.
