# Supplementary material for: Persistent TFIIH binding to non-excised DNA damage causes cell and developmental failure
Source: Nat Commun. 2024 Apr 25;15:3490. doi: 10.1038/s41467-024-47935-9 (PMC11045817; doi:10.1038/s41467-024-47935-9)

Related to Figure 2A

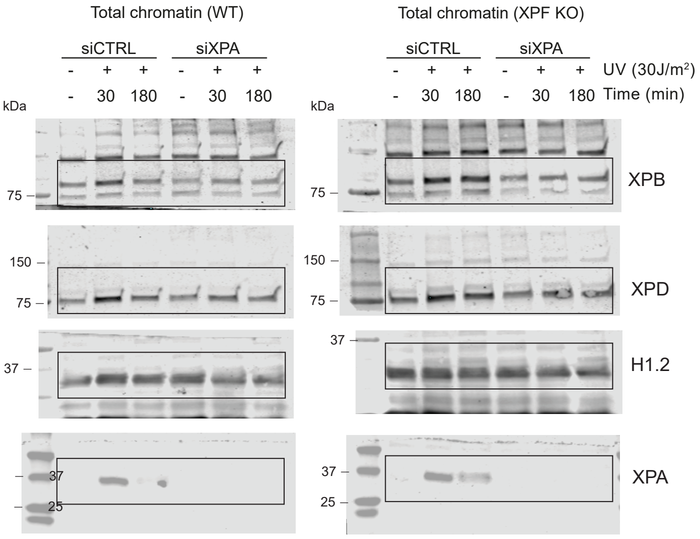

Related to Supplementary Figure 1B

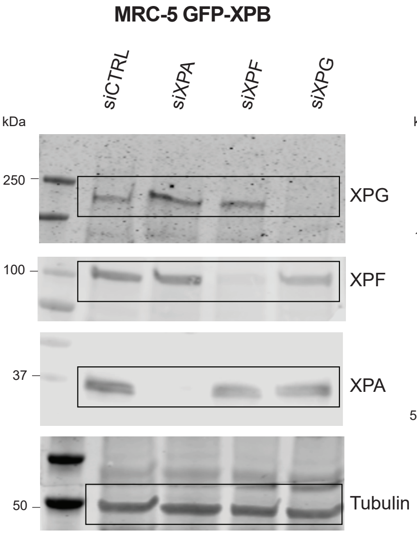

Related to Supplementary Figure 1D

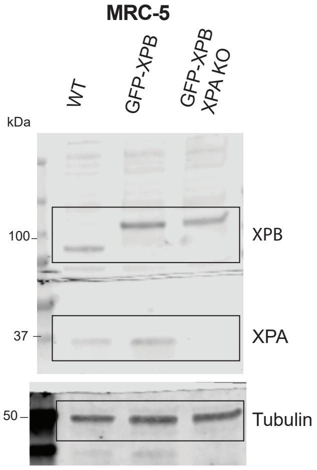

Related to Supplementary Figure 1F

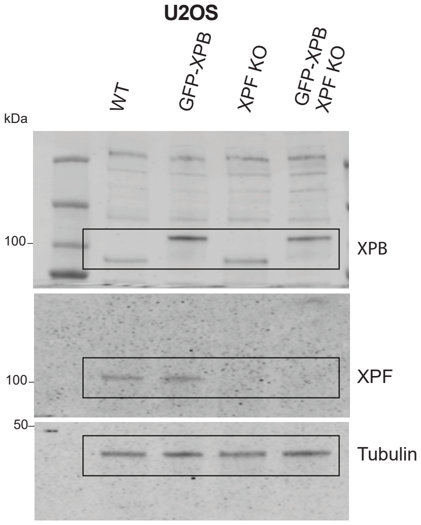

Related to Supplementary Figure 1G

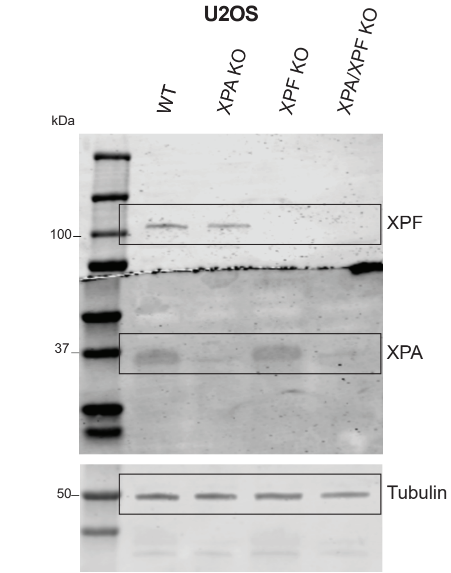

Related to Supplementary Figure 2A

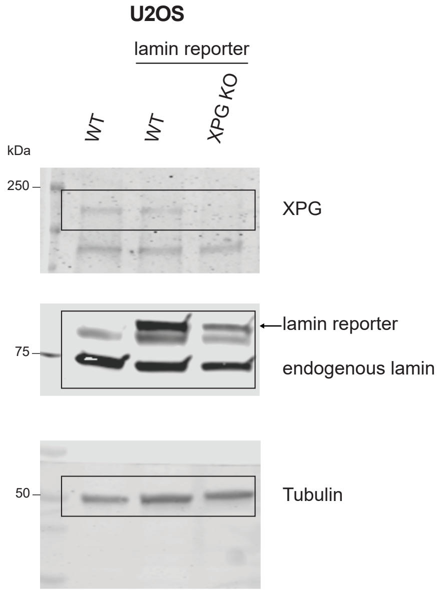

Supplement: Supplementary file 4 — Source Data [file 41467_2024_47935_MOESM4_ESM.zip › original_images_Source_data.pdf]
